# Nicotine Alters Estrogen Receptor-Beta-Regulated Inflammasome Activity and Exacerbates Ischemic Brain Damage in Female Rats

**DOI:** 10.3390/ijms19051330

**Published:** 2018-04-30

**Authors:** Nathan D. d’Adesky, Juan Pablo de Rivero Vaccari, Pallab Bhattacharya, Marc Schatz, Miguel A. Perez-Pinzon, Helen M. Bramlett, Ami P. Raval

**Affiliations:** 1 Cerebral Vascular Disease Research Center, Department of Neurology and Neuroscience Program (D4-5), P.O. Box 016960, University of Miami School of Medicine, Miami, FL 33101, USA; nathandadesky@gmail.com (N.D.d.); pbhattachary@gmail.com (P.B.); marc.schatz@med.miami.edu (M.S.); perezpinzon@med.miami.edu (M.A.P.-P.); 2 Department of Neurological Surgery, The Miami Project to Cure Paralysis, University of Miami School of Medicine, Miami, FL 33136, USA; jderivero@med.miami.edu (J.P.d.R.V.); hbramlett@med.miami.edu (H.M.B.); 3 Bruce W. Carter Department of Veterans Affairs Medical Center, Miami, FL 33125, USA

**Keywords:** stroke, inflammasome, nicotine, estrogen, smoking, women’s health

## Abstract

Smoking is a preventable risk factor for stroke and smoking-derived nicotine exacerbates post-ischemic damage via inhibition of estrogen receptor beta (ER-β) signaling in the brain of female rats. ER-β regulates inflammasome activation in the brain. Therefore, we hypothesized that chronic nicotine exposure activates the inflammasome in the brain, thus exacerbating ischemic brain damage in female rats. To test this hypothesis, adult female Sprague-Dawley rats (6–7 months old) were exposed to nicotine (4.5 mg/kg/day) or saline for 16 days. Subsequently, brain tissue was collected for immunoblot analysis. In addition, another set of rats underwent transient middle cerebral artery occlusion (tMCAO; 90 min) with or without nicotine exposure. One month after tMCAO, histopathological analysis revealed a significant increase in infarct volume in the nicotine-treated group (64.24 ± 7.3 mm^3^; mean ± SEM; *n* = 6) compared to the saline-treated group (37.12 ± 7.37 mm^3^; *n* = 7, *p* < 0.05). Immunoblot analysis indicated that nicotine increased cortical protein levels of caspase-1, apoptosis-associated speck-like protein containing a CARD (ASC) and pro-inflammatory cytokines interleukin (IL)-1β by 88% (*p* < 0.05), 48% (*p* < 0.05) and 149% (*p* < 0.05), respectively, when compared to the saline-treated group. Next, using an in vitro model of ischemia in organotypic slice cultures, we tested the hypothesis that inhibition of nicotine-induced inflammasome activation improves post-ischemic neuronal survival. Accordingly, slices were exposed to nicotine (100 ng/mL; 14–16 days) or saline, followed by treatment with the inflammasome inhibitor isoliquiritigenin (ILG; 24 h) prior to oxygen-glucose deprivation (OGD; 45 min). Quantification of neuronal death demonstrated that inflammasome inhibition significantly decreased nicotine-induced ischemic neuronal death. Overall, this study shows that chronic nicotine exposure exacerbates ischemic brain damage via activation of the inflammasome in the brain of female rats.

## 1. Introduction

Cigarette smoking is a preventable risk factor for stroke, and smoking-ingested nicotine exacerbates post-stroke brain damage [1,2]. Stroke disproportionately kills more women than men and remains one of the leading causes of death and disability in the U.S. Women have a higher risk of stroke, as well as a higher mortality rate associated with stroke, and a higher tendency for more frequent recurrent strokes than men [3,4,5].

Although we know that women are more susceptible to stroke, we have a limited understanding of the underlying mechanisms for increased stroke severity and possible sex-specific prevention and treatment of stroke. Sex differences in stroke are highly complicated, and sex-specific risk factors in part account for epidemiological findings in stroke incidence, prevalence, and mortality. As stated in a review by Girijala et al., there are a number of modifiable risk factors such as cardiac conditions, hypertension, diabetes mellitus type 2, metabolic syndrome, alcohol consumption, physical inactivity, and cigarette smoke exposure, which are common for both sexes [4,6,7]. At the same time, there are female specific risk factors, such as pregnancy, pre-eclampsia, gestational diabetes, migraine with aura during pregnancy, oral contraceptive (OC) use, menopause, and hormone replacement therapy (HRT), which make women more susceptible to stroke [4,8]. What is more striking is that even among women there are studies that clearly link more damaging effects of stroke in women who combine OC/HRT and cigarette smoking. This also points to the facts that: (1) OC/HRT and cigarette smoking/tobacco use have synergistic deleterious effects on a woman’s brain and (2) a gender non-specific risk factor of cigarette smoking has unique effects on the female brain that need to be identified and targeted to reduce consequences of stroke in women. Cigarette smoking, however, is an important modifiable risk factor that an individual does have control over. Nicotine, the main active ingredient in tobacco, has been shown to aggravate ischemic brain damage [9], and the mechanism by which nicotine contributes to poor outcomes after cerebral ischemia is yet to be fully elucidated.

Smoking-attributed nicotine is known to inhibit aromatase enzyme activity, which catalyzes the conversion of androgens into estrogens [10]. Consequently, nicotine reduces circulating estrogen levels and leads to early onset of menopause in women [11,12,13,14,15,16,17,18,19]. In laboratory studies on female rats, we confirmed the aforementioned epidemiological findings that chronic nicotine exposure reduced endogenous 17β-estradiol (E_2_; a potent estrogen) levels [9]. Estrogen-mediated neuroprotection requires activation of estrogen receptor-alpha (ER-α) and beta (ER-β). Silencing of hippocampal ER-β but not ER-α, abolishes E_2_-induced ischemic protection, suggesting a key role of ER-α and/or ER-β-activation [20,21,22]. Our study demonstrated that ER-β activation regulates inflammasome activation. The inflammasome is an arm of the innate immune response involved in the activation of the pro-inflammatory cytokines interleukin (IL)-1β and IL-18 through the processing of caspase-1 [23]. The inflammasome is comprised of the signaling proteins caspase-1 and apoptosis-associated speck-like protein containing a CARD (ASC) [23]. In a published study, we demonstrated that silencing of ER-β attenuated E_2_-mediated decrease in caspase-1, ASC and IL-1β [24]. On the other hand, ER-β agonist treatment reduces inflammasome activation and ischemic damage in reproductively senescent female rats. ER-β agonist treatment significantly decreased inflammasome activation and increased post-ischemic neuronal counts by 32% (*p* < 0.05), as compared to the vehicle-treated, reproductively senescent rats [24]. Studies from our laboratory also showed that chronic nicotine exposure decreased membrane-bound and mitochondrial ER-β, but not ER-α protein levels in the brain [9,25]. Therefore, in the current study, we hypothesized that chronic nicotine exposure activates the inflammasome in the brain, thus exacerbating ischemic brain damage in female rats.

## 2. Results

### 2.1. Nicotine Reduces ER-β Protein Levels in the Brain of Female Rats

**Figure 1:**
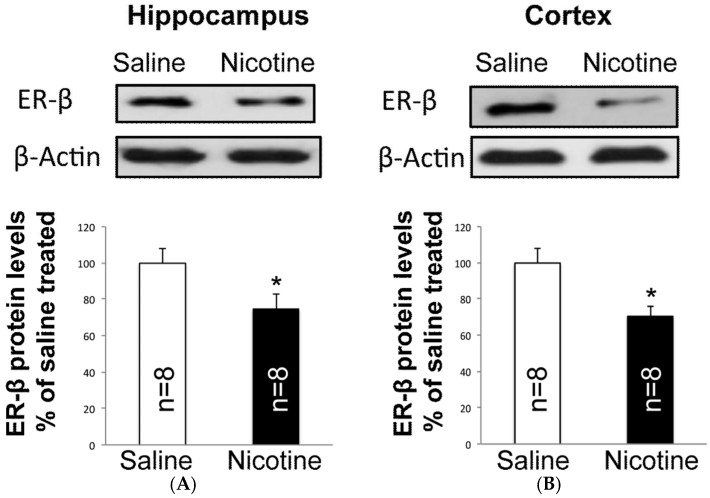
Nicotine decreases estrogen receptor beta (ER-β) protein expression in the hippocampus and cortex of female rats. Immunoblot analyses show significant reduction in the ER-β proteins of nicotine treated (**A**) hippocampus and (**B**) cortex when compared to the saline group. Data are presented mean ± SEM (* *p* < 0.05), *n* = 8.

Because our previous results demonstrated that nicotine reduced the level of membrane-bound and mitochondrial ER-β in the hippocampus, in this study, we investigated protein expression of ER-β in the cortex, the main brain area vulnerable after transient middle cerebral artery occlusion (tMCAO). Our results demonstrated that nicotine significantly reduced cortical ER-β protein levels as compared with the saline group (Figure 1). ER-β protein levels after nicotine showed a 30% (*n* = 8; *p* < 0.05) and 31% (*n* = 8; *p* < 0.05) reduction in cortex and hippocampus, respectively, as compared with saline (100%; *n* = 8).

### 2.2. Nicotine Increases Inflammasome Activation in the Brain of Female Rats

**Figure 2:**
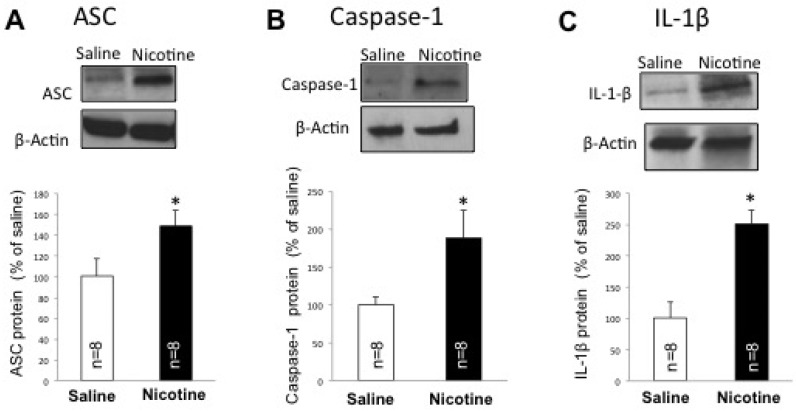
Nicotine increases inflammasome protein expression in the cortex of female rats. Immunoblot analyses show an increase in the inflammasome proteins (**A**) Caspase-1 (**B**) apoptosis-associated speck-like protein containing a CARD (ASC), and (**C**) IL-1β when compared to the saline group. Data are presented mean ± SEM (* *p* < 0.05), *n* = 8.

Since nicotine is an immunomodulatory agent and inflammasome activation plays a key role in ischemic brain damage, we then tested whether nicotine alters inflammasome protein expression. We obtained protein lysates from the cortex of female rats exposed to nicotine and resolved them by immunoblot analysis for the expression of active caspase-1, ASC and IL-1β. Our findings indicate a significant increase in these inflammasome proteins (Figure 2). Accordingly, nicotine increased protein levels of caspase-1, ASC and IL-1β by 88% (*p* < 0.05), 48% (*p* < 0.05) and 149% (*p* < 0.05) respectively in the cortex, as compared to the saline-treated group (Figure 2).

### 2.3. Nicotine Worsens Infarct Volume and Neurodeficit Score after tMCAO

**Figure 3:**
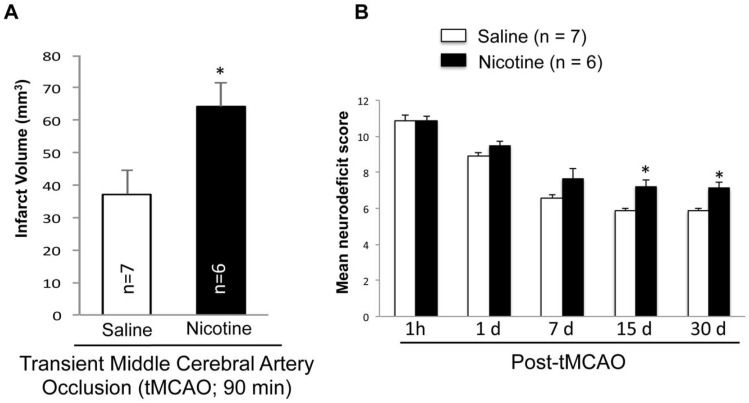
Nicotine increases infarct volume after tMCAO. (**A**) Infarct volume was measured by analyzing lesions after rats were subjected to 90 min of tMCAO. Volume was measured in mm^3^. (**B**) Neurodeficit score was measured in rats post tMCAO. Higher scores represent a greater neurodeficit. Nicotine-treated rats showed significantly higher neurodeficit scores after ischemia when compared to control. Data presented are mean ± SEM (* *p* < 0.05)

Since inflammasome increase in the brain can exacerbate post-stroke outcomes, we tested the hypothesis that chronic nicotine exposure exacerbates stroke outcomes. Rats exposed to nicotine or saline for 16 days underwent tMCAO and were allowed to recover for 30 days before histological analysis. Our data demonstrated significantly higher mean infarct volume in the nicotine-treated group (64.24 ± 7.3 mm^3^; *n* = 6) compared to the saline-treated group (37.12 ± 7.37 mm^3^; Mean ± SEM; *n* = 7, *p* < 0.05) (Figure 3A). Histological analysis of nicotine- or saline-treated rat brains that underwent sham surgery did not show any infarct. Figure 3B shows that the neurodeficit score in each group was more than 10 when tested at 1 h after tMCAO. Neurodeficit scores 1 h, 1, 7, 15 and 30 days after tMCAO are shown in Figure 3B. The neurodeficit score remained unchanged in nicotine-treated animals. These results indicate that nicotine worsens outcome after stroke.

### 2.4. Inhibition of Inflammasome Activation Decreases Neuronal Cell Death in an In Vitro Model of Stroke

**Figure 4:**
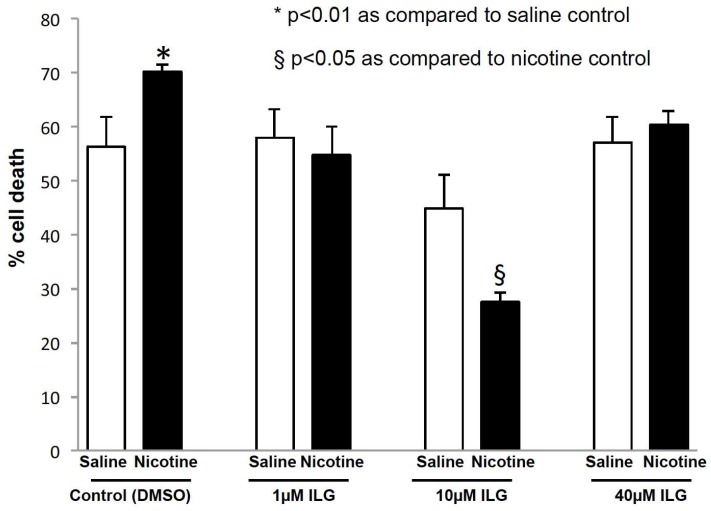
ILG decreases cell death in hippocampal organotypic slice cultures after oxygen-glucose deprivation (OGD). PI fluorescence, which represents cell death, was measured in organotypic brain slices exposed to nicotine or saline treatment, then ILG at varying concentrations 24 h before OGD. Data shown represent cell death normalized to the respective saline groups. To normalize data, the mean of the subgroup was divided by its respective control. PI fluorescence was significantly stronger in the nicotine group than the saline group. PI fluorescence significantly decreased nicotine induced cell death in the nicotine group treated with 10 µM ILG, as compared to the nicotine control. Data are presented mean ± SEM (* *p* < 0.01, § *p* < 0.05). *n* = 4 to 11 per group.

Since chronic nicotine exposure increases inflammasome proteins in the brain of female rats, as a proof-of-principle, we tested the hypothesis that the inhibition of nicotine-induced inflammasome activation improves post-ischemic neuronal survival using our well-established in vitro model of cerebral ischemia. We tested this hypothesis in organotypic cultures by exposing slices to saline or nicotine for 14–19 days. We found that nicotine significantly increased ischemic neuronal death in the Cornu Ammonis area 1 (CA1) region of the hippocampus as compared to saline. The propidium iodide (PI) fluorescence values were 56 ± 5.5% (*n* = 10) and 70 ± 1.2% (*n* = 10) in the saline and nicotine + vehicle-control groups respectively (*p* < 0.01). Nicotine and saline exposed slices were treated with the inflammasome inhibitor isoliquiritigenin (ILG; 1, 10, and 40 µM). Inflammasome inhibition at 10 µM ILG concentration showed a significant decrease in nicotine-induced ischemic neuronal death when compared to the nicotine + vehicle group. The PI fluorescence values of the saline + ILG, nicotine + ILG, and vehicle treated nicotine groups were 44 ± 6.2% (*n* = 8), 27 ± 1.5% (*n* = 8), and 70 ± 1.2% (*n* = 10; *p* < 0.05), respectively. The PI fluorescence values of the nicotine + 1 µM ILG and nicotine + 40 µM ILG were 55 ± 5.3% (*n* = 8) and 61 ± 2.5% (*n* = 8), which were not significantly different as compared to PI values of saline + 1 µM ILG and saline + 40 µM ILG groups (Figure 4).

## 3. Discussion

This study revealed for the first time that chronic nicotine exposure increases inflammasome activation in the brain and exacerbates post-ischemic damage in the brain of female rats. Our study also demonstrated that inhibition of inflammasome activation specifically attenuates nicotine-induced ischemic cell death in an in vitro model of ischemia. Inhibition of inflammasome activation was achieved with ILG, which is a chalconoid found in licorice. Studies have found that ILG inhibits inflammasome activation via the inhibition of ASC oligomerization and the inhibition of nucleotide-binding oligomerization domain (NOD)-like receptor 3 (NLRP3) activation [26,27]. In our study, nicotine-exposed hippocampal slice-cultures were treated with ILG, one day prior to the induction of OGD. Therefore, the observed neuroprotective effect of ILG in our study suggests the suppression of nicotine-induced inflammasome activation in the brain. In vivo the half-life on ILG has been identified to be around 4 h [28]. In this study, ILG was added before and during OGD. In vitro, when ILG was applied to liver microsomes, the half-life was found to be 25.3 min. Since brain cells do not have the clearance capacity of liver cells, we estimate that the half-life of ILG in the brain slices is greater. Importantly, since OGD in our study was done in the presence of ILG for 45 min, we anticipate that for all or most of the duration of the experiment, ILG was present. At the low dose, the concentration of 1 µM ILG was not enough to prevent cell death. However, at the middle dose of 10 µM we found the concentration optimum at which ILG can prevent cell death in both the saline as well as the nicotine groups. The ILG treatment showed more rigorous neuroprotection in nicotine treated group as compared to saline group, which suggests that effects of ILG might wean off prior to post-OGD inflammasome activation and observed effects of ILG in nicotine group could be outcome of pre-OGD inhibition of ASC. Although the mechanism of action of how ILG inhibits inflammasome activation remains unknown, it has been shown that this inhibition occurs between 1 and 10 µM [29], and that at the higher dosages of even 30 µM ILG does not inhibit the inflammasome. Thus, it is possible that at the 40 µM the effects of ILG on inflammasome inhibition are absent, resulting in greater cell death in the 40 µM similar to the 1 µM group.

It has been shown that inflammasome activation in the brain is regulated by sex hormones [24]. In a previous study from our laboratory, we reported that ER-β attenuates inflammasome activation in the brain of female rats. Specifically, silencing of hippocampal ER-β increased caspase-1, ASC, and the pro-inflammatory cytokine interleukin-1β (IL-1β) in the hippocampus of ovariectomized rats. Conversely, periodic activation of ER-β significantly decreased inflammasome activation and reduced post-ischemic neuronal death in the hippocampus of reproductively senescent female rats. It has been shown that aging as well as long-term nicotine usage reduces ER-β availability in the brain [30,31]. Therefore, nicotine-induced inflammasome activation in the brain could be due to the direct immunomodulatory nature of nicotine or the loss of ER-β following long-term nicotine exposure in the brain. The latter scenario is consistent with females having a higher risk from nicotine toxicity. This notion is supported by a study showing that hippocampal neuronal damage is greater in female rats following chronic nicotine exposure [27].

Inflammasome activation occurs after the detection of pro-inflammatory molecules by pattern recognition receptors, such as a toll-like receptor or a NOD-like receptor (NLR) [32]. The mechanisms of inflammasome activation include the generation of mitochondrial reactive oxygen species (ROS) and the translocation of NLRP3 to the mitochondria [33]. Interestingly, the nicotinic acetylcholine receptor (7α-nAChR) has been found in neuronal mitochondria [34] and its physiological significance remains unknown. However, it is likely that it is involved in buffering cytoplasmic calcium [35,36]. It is through the mechanism of calcium buffering in which one study found that 7α-nAChRs located in the mitochondria regulates inflammasome activation in peritoneal mouse macrophages [37]. It is well documented that calcium overload can trigger mitochondrial dysfunction, promote the production of reactive oxygen species (ROS), and disrupt the mitochondrial permeability transition pore, eventually leading to neuronal death [38,39,40]. Therefore, nicotine-induced mitochondrial dysfunction, which has been described in several studies [41,42,43], may be responsible for the increase in stroke volume in female rats. However, studies are needed to understand the upstream mechanisms leading to mitochondrial dysfunction that increases the susceptibility to ischemia.

Finally, relinquishing the smoking habit reduces the risk for stroke; however, impact of smoking cessation on stroke outcome remains unknown. Because it is difficult to stop smoking, more tobacco users have switched to the “fashionable” e-Cigarette (electronic nicotine delivery systems) as an alternative to tobacco smoking or even as an aid for smoking cessation. However, the safety of e-Cigarettes remains questionable and as demonstrated in the current study, negative effects of nicotine on brain will persist, which makes current research in this area timely and of a high impact.

## 4. Materials and Methods

### 4.1. Animals

All animal procedures were carried out in accordance with the Guide for the Care and Use of Laboratory Animals published by the U.S. Public Health and procedures involving animal subjects and were approved by the Animal Care and Use Committee (#A-3224-01, effective 24 November 2015) of the University of Miami.

### 4.2. In Vivo

Adult female Sprague–Dawley rats (290 ± 20 g; 6–7 months old) were used for this study. The stages of estrous cycle were monitored as described [44] and only rats showing at least three consecutive normal (4 day) estrous cycles were used for our experiments.

### 4.3. Nicotine or Saline Treatment

To obtain sustained nicotine delivery, osmotic pumps (type 2ML2, Alzet Corp., Palo Alto, CA, USA) containing nicotine or saline were implanted in rats for 16–21 days as described [30]. The pump delivered a fixed and continuous dose of nicotine hydrogen tartrate (4.5 mg/kg/day, equivalent to 1.5 mg/kg/day free base) throughout the 16–21 days. Rats exposed to nicotine (*n* = 21) or saline (*n* = 20) were divided into two groups. One group of eight rats treated with either nicotine or saline was used for brain tissue collection, while the remaining rats in both treatment groups underwent transient middle cerebral artery occlusion (tMCAO) or sham surgery. Since we have previously demonstrated that the higher endogenous estrogen levels seen during proestrus protect CA1 neurons against cerebral ischemia [45], tMCAO or tissue collection was performed only when nicotine/saline treated rats were in proestrus stage. In those instances where rats were not in the proestrus stage on the last scheduled day of treatment, we extended the treatment by 1 or 2 days.

### 4.4. Transient Middle Cerebral Artery Occlusion (tMCAO) and Infarct Volume

Nicotine or saline treated rats were exposed to ischemic stroke (90 min) using an occluding intraluminal suture inserted past the internal carotid artery to occlude the middle cerebral artery as described previously [46,47]. In parallel, we also performed sham surgery on nicotine or saline treated rats. During this sham procedure rats were exposed to anesthesia for a period similar to that of the tMCAO group. During the surgical procedure of tMCAO or sham, physiological parameters including pCO_2_, pO_2_, pH, HCO_3_ and arterial blood pressure were maintained within normal limits prior to and after tMCAO (data presented as Table 1). Body and head temperatures were maintained at 37 ± 0.2 °C throughout the experiment with assistance of lamps placed above the animal’s body and head. One month after tMCAO surgery, rats were perfused with saline, then with FAM (a mixture of formaldehyde, glacial acetic acid, and methanol; 10:10:80), and brains were prepared, sectioned, and stained to obtain infarct volumes. The sample preparations and procedures are described in more detail in our previous publication [48]. Brains were then embedded with paraffin and 10 µm thick sections were obtained. Infarct volume measurements were adapted from previous publications [49,50]. Briefly, the same nine coronal sections were selected for every animal (Bregma levels 5.2, 2.7, 1.2, −0.3, −1.3, −1.8, −3.8, −5, −7.3). Infarcted area was then measured on each section using MCID software (version, Manufacturer, City, US State abbrev. if applicable, Country). Infarct volume was then determined similar to previously used methods [49,50].

### 4.5. Neurodeficit Scoring

The neurological score was monitored at an hour, 1, 7, 15 and 30 days after tMCAO. A standardized neurobehavioral test battery was conducted as described previously [51], which includes tests for postural reflex, sensorimotor integration, and proprioception. Total neurological score ranged from a normal score of 0 to a maximal possible score of 12.

### 4.6. Western Blotting

Rats exposed to nicotine (16–21 days) or saline were anesthetized using 5% isoflurane, decapitated, and the hippocampal and cortical tissues were collected, flash frozen, and stored at −80 °C. At the time of immunoblotting, hippocampal and cortical tissues were homogenized, protein content was analyzed, and proteins were separated by 12% SDS-PAGE as described [47]. Proteins were transferred to Immobilon-P (Millipore, Burlington, MA, USA) membrane and incubated with primary antibodies against rabbit polyclonal anti-ER-β (1:500; Santa Cruz Biotechnology, Dallas, TX, USA), IL-1β (1:1000; Cell Signaling, Danvers, MA, USA), ASC (1:1000; Santa Cruz Biotechnology), Caspase-1 (1:1000; Novus Biologicals, Littleton, CO, USA), and β-Actin (1:5000; Sigma, St. Louis, MO, USA). All data were normalized to β-Actin (monoclonal; 1:1000; Sigma). Immunoblot images were digitized and subjected to densitometric analysis [45].

### 4.7. In Vitro Organotypic Slice Cultures and Oxygen-Glucose Deprivation

Hippocampal organotypic slice cultures were prepared from female neonatal (9–11 days old) Sprague–Dawley rats and the details of slice culture are as described previously [46,52,53,54]. Briefly, hippocampal slices were cultured for 14–20 days followed by exposure to nicotine (100 ng/mL) or saline (vehicle) for 14–16 days. At the end of the treatment period (~29–37 days), a subgroup of slices (either treated with nicotine or saline) were exposed to the inflammasome inhibitor isoliquiritigenin (ILG; Invivogen, San Diego, CA, USA) at different concentrations (1, 10, or 40 µM) dissolved in dimethyl sulfoxide (DMSO; Sigma-Aldrich, St. Louis, MO, USA) or vehicle control DMSO (1 µL/mL of medium) for 24 h. After inhibitor/control treatment, each subgroup was exposed to OGD (45 min) as described [46,52,53,54]. To determine the extent of neuronal damage following OGD, we used the propidium iodide (PI) method [46,52,53,54]. Briefly, slices were incubated in culture medium supplemented with 2 µg/mL PI (Sigma, St. Louis, MO, USA) for 1 h. Images were taken using an inverted fluorescence microscope. Images of the cultured slices were taken (1) at baseline prior to the ‘test’ ischemia procedure; (2) 24 h after the ‘test’ ischemic insult to assess ischemic damage; and (3) 24 h after *N*-methyl-d-aspartate (NMDA) treatment to assess maximum damage to neuronal cells. The hippocampal CA1 subfield was chosen as the region of interest, and quantification was performed using Scion Image software [46,52,53,54]. The percentage of relative optical intensity (ROI) served as an index of neuronal cell death [46,52,53,54].

### 4.8. Statistical Analysis

The data are presented as mean value ± SEM and results were analyzed by a two-tailed Student’s *t* test and *p* < 0.05 was considered statistically significant.

## Figures and Tables

**Table 1:** Physiological variable. Physiological variables were recorded during the period when the animal was under anesthesia. Rows with bold background denote values after tMCAO. Mean arterial blood pressure (MABP).

Groups	Glucose	pH	pCO_2_	pO_2_	MABP	Post-tMCAO Mortality
Saline + Sham (*n* = 4)	131.0 ± 7.4	7.39 ± 0.5	36.3 ± 2.3	135 ± 35	132.4 ± 6	0
		**7.39 ± 0.4**	**36.0 ± 4.1**	**133 ± 15**	**134 ± 11.7**	
Nicotine + Sham (*n* = 5)	126.4 ± 2.8	7.36 ± 0.4	34 ± 7.2	133 ± 37	135 ± 3	0
		**7.37 ± 0.04**	**38.0 ± 3.4**	**127 ± 28**	**135 ± 11.4**	
Saline + tMCAO (*n* = 8)	130.3 ± 8.7	7.36 ± 0.4	35.3 ± 2.3	134 ± 39	131 ± 7.7	1
		**7.37 ± 0.05**	**34.3 ± 5.2**	**140 ± 15**	**134 ± 9.3**	
Nicotine + tMCAO (*n* = 8)	127.4 ± 6.8	7.4 ± 0.2	38.08±4.5	130 ± 35	129 ± 7.8	2
		**7.39 ± 0.04**	**35.8 ± 3.9**	**138 ± 25**	**132 ± 5.3**	

## Abbreviations

ASC	Apoptosis associated speck-like protein containing a caspase recruitment domain
BBB	Blood brain barrier
DMSO	Dimethyl sulfoxide
e-Cigarette	Electronic cigarettes
ER-β	Estrogen receptor subtype beta
FAM	Formaldehyde, glacial acetic acid, and methanol
IL-1β	Interleukin-1β
ILG	Isoliquiritigenin
nAChR	Nicotinic acetylcholine receptor
NLR	NOD-like receptor
NMDA	*N*-methyl-d-aspartate
OGD	Oxygen-glucose deprivation
PI	Propidium iodide
tMCAO	Transient middle cerebral artery occlusion
TNF	Tumor necrosis factor
